# Efficacy of Computer-Aided Static Navigation Technique on the Accuracy of Endodontic Microsurgery. A Systematic Review and Meta-Analysis

**DOI:** 10.3390/jcm10020313

**Published:** 2021-01-15

**Authors:** Álvaro Zubizarreta-Macho, César Castillo-Amature, José María Montiel-Company, Jesús Mena-Álvarez

**Affiliations:** 1 Department of Endodontics, Faculty of Health Sciences, Alfonso X El Sabio University, 28691 Madrid, Spain; amacho@uax.es (Á.Z.-M.); od.castilloendo@gmail.com (C.C.-A.); 2 Department of Stomatology, Faculty of Medicine and Dentistry, University of Valencia, 46010 Valencia, Spain; jose.maria.montiel@uv.es

**Keywords:** apicoectomy, endodontic surgery, apex root-end location, static navigation, computer-aided technique

## Abstract

The aim of this systematic review and meta-analysis was to analyze the efficacy of the computer-aided static navigation technique on the accuracy of root apex location in endodontic microsurgery. Material and Methods: A systematic literature review and meta-analysis, based on Preferred Reporting Items for Systematic Reviews and Meta-Analyses (PRISMA) recommendations, of clinical studies that evaluated the apex location rate of the computer-aided static navigation techniques applied to endodontic microsurgery. A total of four databases were consulted in the literature search: Pubmed-Medline, Scopus, Cochrane, and Web of Science. After eliminating duplicated articles and applying the inclusion criteria, seven articles were selected for the qualitative and the quantitative analysis. Results: The root apex location success rate stated at 96.8% (confidence interval (CI): 93.0–100%) of the cases performed through a computer-aided static navigation technique. The prediction interval ranges from 91.4% to 100%. The meta-analysis did not detect heterogeneity between the combined studies (Q-test = 6.15; *p*-value = 0.407; I2 = 2.4%). The computer-aided static navigation techniques showed a root apex location success rate 27 times higher than conventional endodontic microsurgery procedures (Q test = 0.80; *p* = 0.671; I2 = 0%). Three studies of computer-aided static navigation techniques and control group were compared using a random effects model with the Mantel-Haenszel method with a statistically significant odds success ratio of 27.7, with a 95% confidence interval between 11.3 and 68.1 (z test = 7.23; *p* < 0.0001). Conclusions: According to in vitro studies analyzed, endodontic microsurgeries performed through computer-aided static navigation techniques show a high precision.

## 1. Introduction

Bacterial infection is considered the main factor in establishing the effects of pulp tissues, which may lead to subsequent irreversible pulp damage, necrosis, and the formation of periapical lesions [1]. Complete removal or significant reduction of the bacterial load present in the root canal system during root canal treatment is considered an essential factor to determine the long-term outcome of the root canal treatment. Development of apical periodontitis has been reported in 44.9% of studied cases [2], mainly related to persistent or secondary endodontic infections; however, according to the Toronto study, 4–6 years after endodontic initial treatment, 86% of teeth healed and 95% remained asymptomatic and functional [3]. Non-surgical endodontic retreatment is recommended in root canal treatment failure; however, the non-surgical endodontic retreatment presents a success rate of 80% [4]. Endodontic microsurgery procedures have been recommended after unsuccessful non-surgical endodontic retreatment, when non-surgical endodontic retreatment is impossible or has an unfavorable prognosis [5], when orthograde access to the apical portion of the root canal system is not effective or technically possible. The reasons may vary from unnecessary removal of a sound coronal restoration or irreparable damage, such as fracture, during disassembly of an extensive post and crown prosthesis [6]. Endodontic microsurgery procedures comprise the removal of necrotic and infected periapical tissues, resection of the apical part of the tooth (apicoectomy), and preparation of the root-end cavity for the insertion of a retrograde filling material [7]. The outcome of conventional endodontic microsurgery has reported 90% complete healing of periapical tissues [8]; however, the inaccuracy of root apex location may cause intraoperative complications as root perforation, maxillary sinus affection, or weakened dental structure [4]. The development of advanced radiodiagnostic techniques as the cone beam computed tomography (CBCT) exam has allowed a better diagnosis and planning of the endodontic microsurgery and root apex location, improving the endodontic surgery success rate [9]. In addition, the development of the computer-aided static (SN) navigation technique allows a drilling guidance during the endodontic microsurgery procedures [10], improving the accuracy of root apex location and allowing an accurate and safe endodontic microsurgery, especially in posterior and lingual/palatal roots with compromised access or location [4,10].

The computer-aided static (SN) navigation method involves the application of a surgical template to guide osteotomy and facilitate precise apex localization in endodontic microsurgery. After CBCT imaging and cast scan data are transferred to implant surgical planning software, the data are superimposed. The surgical template is then printed using a three-dimensional printer. Endodontic microsurgery includes application of this printed surgical template. A computer-aided design/computer-aided manufacturing (CAD/CAM)-guided surgical template minimizes the extent of osteotomy and enables precise targeting of the apex. The aim of this systematic review and network meta-analysis was to analyze the efficacy of the computer-aided static navigation technique on the accuracy of root apex location in endodontic microsurgery by means of a systematic review and meta-analysis, with a null hypothesis (H0) stating that there would be no differences between the computer-aided static navigation technique and the conventional apicoectomy procedure on the accuracy of root apex location in endodontic microsurgery.

## 2. Materials and Methods

### Study Design

This bibliographic search was conducted following PRISMA (Preferred Reporting Items for Systemic Reviews and Meta-Analyses, http://www.prisma-statement.org) guidelines for systematic reviews and meta-analyses (PROSPERO registration number: CRD42020192264). The review also fulfilled the PRISMA 2009 Checklist [11]. The PICO (population, intervention, comparison, and outcome) question was, ‘Which is efficacy of computer-aided static navigation techniques on the accuracy of endodontic microsurgery?’ with the following components: population: teeth submitted to endodontic microsurgery performed through computer-aided static navigation techniques; intervention: endodontic microsurgery procedures performed through computer-aided static navigation techniques; comparison: endodontic microsurgery procedures performed through static navigation systems; and outcome: accuracy and apex location by endodontic microsurgery. An electronic search was conducted in the following databases: PubMed, Scopus, Embase, and Web of Sciences. The search covered all the literature published internationally up to April 2020. The search included ten medical subject heading (MeSH) terms: ‘apicoectomy’; ‘endodontic surgery’; ‘endodontic microsurgery’; ‘periradicular surgery’; ‘root-end resection’; ‘guided endodontic surgery’; ‘static navigation’; ‘accuracy’; ‘deviation’; and ‘apex location’. The Boolean operators applied were (‘OR’ and ‘AND’). The search terms were structured as follows: [(‘apicoectomy’) OR (‘endodontic surgery’) OR (‘endodontic microsurgery’) OR (‘periradicular surgery’) OR (‘root-end resection’) OR (‘guided endodontic surgery’) OR (‘static navigation’)] AND [(‘accuracy’) OR (‘deviation’) OR (‘apex location’)]. Two researchers (C.C.A. and J.M.A.) conducted the database searches in duplicate independently. Titles and abstracts were selected applying inclusion and exclusion criteria. One researcher (C.C.A.) extracted data on the relevant variables. The systematic review was carried out by two researchers (A.Z.M. and J.M.A) and subsequent meta-analysis was performed by two researchers not involved in the selection process (A.Z.M. and J.M.M.C.).

Inclusion criteria: Studies recorded in databases as in vitro randomized experimental trial (RET), randomized clinical trials (RCT), clinical trials (CT), and case series (CS) from two teeth. Teeth submitted to endodontic microsurgery through computer-aided static navigation technique. No restriction was placed on the year of publication or language. Exclusion criteria: systematic literature reviews, clinical cases, and editorials. The following data were extracted from each article: author and year of publication; title and journal in which the article was published; sample size (n); and accuracy of apex location. Studies that analyzed static navigation techniques were included in the systematic review and meta-analysis.

The risk of bias of the in vitro studies selected for review was assessed using the Current Research Information System (CRIS) scale for methodological quality assessment. The CRIS scale consists of four items that evaluate the sample preparation and handling, allocation sequence and randomization process, whether the evaluators were blinded, and statistical analysis. Studies with information about all variables were deemed to be of good quality; if two to three variables were present, they were deemed of fair quality; and lastly, they were classified as being of poor quality when none or just one aspect was covered [12]. The risk of bias of the clinical studies selected for review was assessed using the Jadad scale for methodological quality assessment of clinical trials. The Jadad scale consists of five items that evaluate randomization, researcher and patient blinding, and description of losses during follow-up, producing a score of 0–5; scores of less than 3 are considered low quality [13].

The included studies for the meta-analysis were combined using a random effects model with various methods according to the estimated effect size. The inverse method of variance was used to estimate the root apex location success rate, the Mantel-Haenszel method for the odds ratio (OR), and the inverse method of variance for the mean difference. For all the estimated variables, its 95% confidence interval was calculated. Heterogeneity between the combined studies was assessed using the Q test (*p*-value < 0.05) and quantified with the I2, considering a slight heterogeneity if it is between 25 and 50%, moderate between 50 and 75%, and high if >75%. The existence of statistical significance was assessed using the Z test (*p*-value < 0.05). Meta-analyzes were represented with a forest plot. Publication bias was assessed using the trim and fill adjustment method, and represented with funnel plots.

## 3. Results

### 3.1. Flow Diagram

**Figure 1:**
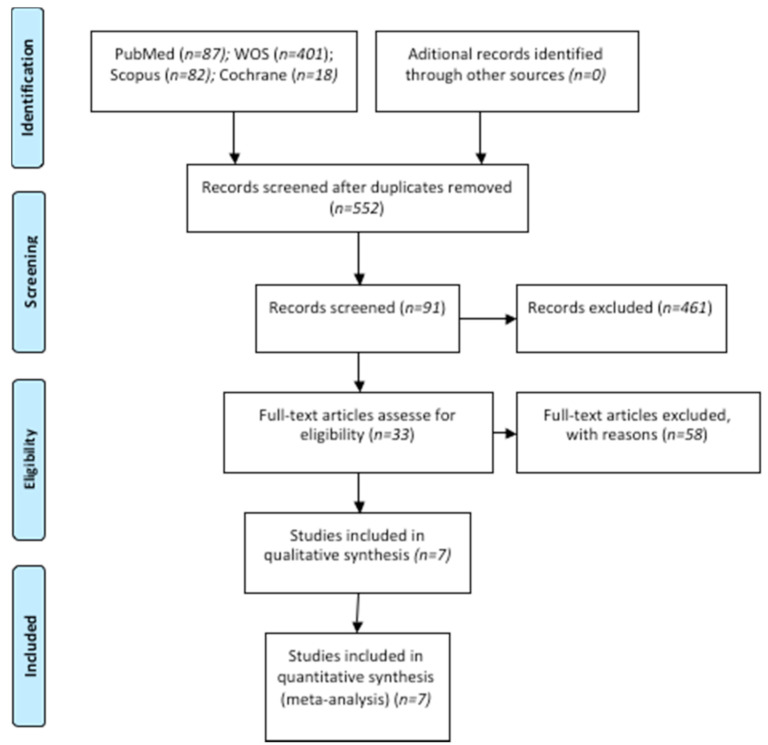
Preferred Reporting Items for Systematic Reviews and Meta-Analyses (PRISMA) flow diagram. WOS, Web of Sciences.

The initial electronic search identified 87 articles in PubMed, 401 in Web of Sciences (WOS), 18 in Cochrane, and 82 in Scopus. Of the total 588 works, 36 were discarded as duplicates. After reading the titles and abstracts, a further 461 were eliminated, leaving a total of 91. A further fifty-eight were rejected as they failed to fulfill the following inclusion criteria: they did not include canal location rate or the minimum sample size. A final total of seven articles were included in the qualitative synthesis. These seven articles were included in the quantitative synthesis as these included all the data and variables required (Figure 1).

### 3.2. Qualitative Analysis

Of the seven articles included, three were experimental trials [14,15,16], three were CS [17,18,19] and one was a clinical trial (CT) [20]. In addition, three studies compared static navigation with regard to conventional endodontic microsurgery [14,15,16]. Experimental trials presented a sample size ranging from 42 in the study by Fan, 2019 [15] to the high figure of 110 in Pinsky’s study, 2007 [16]. However, most clinical studies were CS with two to three patients [17,18,19] and only one CT was included with a sample size of 14 apicoectomies in 13 teeth of 11 patients [16] (Table 1).

### 3.3. Quality Assessment

The results of methodological quality assessment using the CRIS scale are shown in Table 2. Two articles [14,15] obtained scores of 4 and one article obtained the score of 3 [16], indicating high methodological quality.

The results of methodological quality assessment using the Jadad scale are shown in Table 3. The Jadad scale obtained tree articles “not applicable”, because they were a case series [17,18,19], and the authors of these articles did not blind and did not randomize the studies. In addition, the CT study [20] was also described as “not applicable”, because it was not a comparative study and the authors did not randomize the study.

### 3.4. Quantitative Analysis

#### 3.4.1. Root Apex Location Success Rate

**Figure 2:**
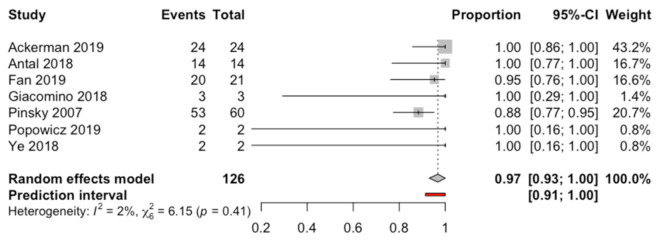
Forest plot of root apex location success rate between the studies selected. CI, confidence interval.

Seven studies were selected and combined using a random effects model with an inverse variance method. The root apex location success rate was stated at 96.8% with a confidence interval between 93.0% and 100% of the cases performed through a computer-aided static navigation technique (Figure 2). The prediction interval ranges from 91.4 to 100%. The meta-analysis did not detect heterogeneity between the combined studies (Q-test = 6.15; *p*-value = 0.407; I^2^ = 2.4%) (Figure 2).

#### 3.4.2. Comparison between Computer-Aided Static Navigation Technique and Control Group

**Figure 3:**
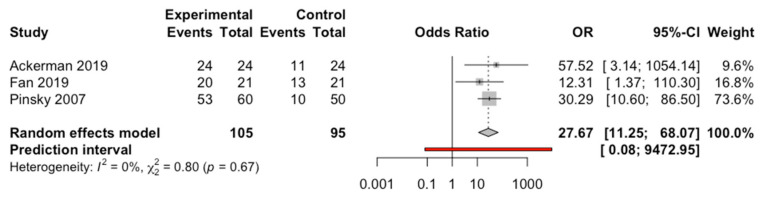
Forest plot of root apex location success odds ratio (OR) between computer-aided static navigation technique and control group.

Three in vitro studies of computer-aided static navigation techniques and control group were compared using a random effects model with the Mantel-Haenszel method with a statistically significant OR of 27.7, with a 95% confidence interval between 11.3 and 68.1 (z test = 7.23; *p* < 0.0001). Root apex location success rate is 27 times higher using computer-aided static navigation techniques. No heterogeneity was detected (Q test = 0.80; *p* = 0.671; I^2^ = 0%) (Figure 3).

**Figure 4:**
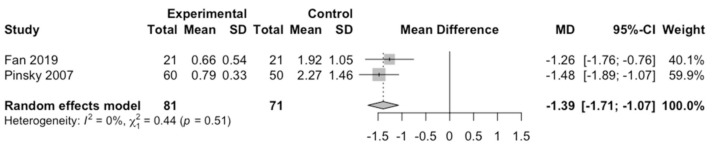
Forest plot from the meta-analysis of the mean difference (MD) in measurement error in the use of a computer-aided static navigation technique with respect to the control group.

Combining two studies using a random effects model with the inverse variance method that measured apex measurement error using a computer-aided static navigation technique with respect to the control group, statistically significant differences were estimated (z test = −8.53; *p* < 0.0001) estimated at −1.39 mm with a 95% confidence interval between −1.71 and −1.07. No heterogeneity was detected (Q test = 0.44; *p* = 0.508; I^2^ = 0%) (Figure 4).

## 3.5. Publication Bias

**Figure 5:**
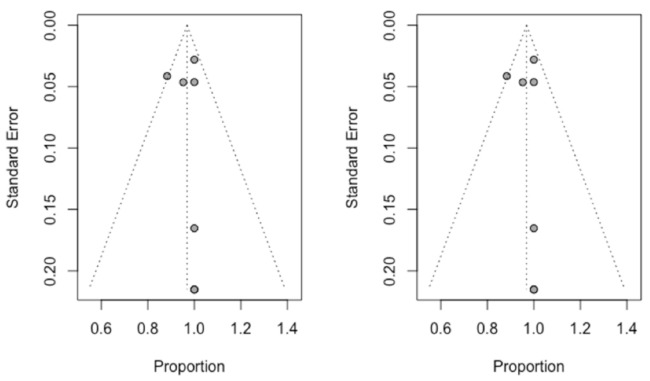
Initial funnel plot and after trim and fill adjustment of the root apex location success rate using a computer-aided static navigation technique.

Used to assess publication bias, the trim and fill method was used to adjust the asymmetry of the funnel plot. No new studies were added to the seven initially combined to obtain a symmetrical image and the estimation of root apex location success rate was not varied, being 96.8% (95% CI between 93% and 100%). Figure 5 shows the two funnel plots (initial and adjusted), indicating a clear absence of publication bias.

## 4. Discussion

The results obtained in the present study accept the null hypothesis (H0) stating that there would be no statistically significant differences between the computer-aided static navigation technique and the conventional apicoectomy procedure on the accuracy of root apex location in endodontic microsurgery.

Computer-aided static navigation procedures were firstly applied to dental implants surgery in order to improve the dental implant placement accuracy associated with the freehand dental implant placement technique and prevent intraoperative complications including cortical or dental perforations and damage to particular anatomical structures, such as the inferior alveolar nerve or the maxillary sinus, due to implant malpositioning [21,22]. The freehand dental implant placement technique has shown higher deviation values with respect to the computer-aided static navigation dental implant placement techniques [23]. In addition, computer-aided static navigation dental implant placement has shown a mean horizontal deviation of 0.99 mm (ranging 0.0 mm to 6.5 mm) at the dental implants platform, a mean horizontal deviation of 1.24 mm (ranging from 0.0 mm to 6.9 mm) at the dental implant apex, and a mean angle deviation of 3.81° (ranging from 0.0° to 24.0°) with respect to the longitudinal axis of dental implants [24]. The safer, more accurate, and conservative approach of guided dental implant placement focused the attention of endodontics on computer-aided static navigation procedures; however, the accuracy of computer-aided static navigation techniques associated with endodontics is a concern because the teeth size requires a high accuracy level. Therefore, in vitro and in vivo studies have been performed in order to analyze the accuracy of computer-aided static navigation techniques. Connert et al. reported a higher root canal location success rate and less substance loss between the endodontic access cavity performed by the computer-aided static navigation technique and freehand endodontic access cavities [25]. Zubizarreta et al. also reported statistical significant differences (*p* ˂ 0.05) between the endodontic access cavities planned and performed by computer-aided static navigation techniques with respect to freehand endodontic access cavities at coronal, apical, and angular deviations [10]. In addition, many authors have used computer-aided static navigation procedures to perform the endodontic access cavity in calcific metamorphosis cases [26,27,28] or dental malformations as dens invaginatus [29,30] or dens evaginatus [31]. Furthermore, computer-aided static navigation techniques have also been used to remove prosthetic fiber post into the root canal system [32,33] and guide prosthetic dental crown preparations [34]. The accuracy of endodontic procedures performed by computer-aided static navigation techniques has led to the application of the computer-aided static navigation technique to endodontic surgery procedures. The root apex location by means of conservative surgical access cavities influences the periapical healing outcome of the bone defect [35], operation time, accuracy, and post-operative discomfort [36], without risks of damaging surrounding structures [16]. Therefore, drilling guidance by means of computer-aided static navigation should be considered especially in cases of compromised surgical access, with limited periapical tissue damage and without loss of cortical plate, despite the limited view of the resected root and difficulty in insertion and orientation of ultrasonic tips along the long axis of the tooth [18].

The root apex location is considered one major challenge during endodontic microsurgery procedures [14]. Traditionally, magnification, illumination, microinstruments, and CBCT scans have been used in endodontic microsurgery procedures to improve the root apex location success rate [17]; however, computer-aided static navigation techniques have shown a root apex location success rate 27 times higher than conventional endodontic microsurgery procedures. In addition, the root apex location success rate was stated at 96.8% with a confidence interval between 93.0% and 100% of the cases performed through a computer-aided static navigation technique; therefore, it is highly recommended to use computer-aided static navigation techniques in order to locate the apical root in endodontic microsurgery procedures. Recently, novel technologies have been used in order to accurately locate the root apex. Gambarini et al. reported a case report using computer-aided dynamic navigation techniques to allow the root apex location in endodontic microsurgery. This technology uses an optical triangulation tracking system comprising stereoscopic motion-tracking cameras guiding the drilling process at the planned angle, pathway, and depth of the osteotomy in real time [37]. It has been widely used in dental implant placement, showing significantly (*p* ˂ 0.05) lower deviation values at the coronal entry point (0.71 ± 0.40 mm), apical endpoint (1.00 ± 0.49 mm), and angular deviation (2.26 ± 1.62°) with respect the freehand dental implant placement technique [38]. In addition, the computer-aided dynamic navigation technique has been introduced into the field of endodontics in an attempt to improve the accuracy of root canal location and avoid potential risks associated with this therapeutic procedure [10]. However, the computer-aided dynamic navigation techniques show a high difficulty of keeping the system display in sight during the endodontic surgery and a long learning curve [39]; therefore, further studies are required to validate the technique in the field of endodontic microsurgery.

This systematic review with meta-analysis has limitations related to the risk of not finding articles related to the selection criteria, although the risk is lower when searching in four databases. Clinical studies presented a poor quality with a score of 0 in Jadad criteria. However, most in vitro studies presented a high quality score between 3 and 4 in CRIS criteria. Furthermore, few randomized studies both clinical and in vitro were included. In addition, only three studies were included with a control group comparison, so further clinical studies better designed and of greater quality are necessary to confirm the results.

## 5. Conclusions

The results obtained in this systematic review and meta-analysis based on in vitro studies show a high precision associated with computer-aided static navigation techniques; however, clinical studies are necessary to contrast these results to widely recommend this technique in endodontic surgery.

## Figures and Tables

**Table 1:** Qualitative analysis of articles included in the systematic review.

Author/Year	Study Type	Sample (*n*)	Measurement Procedure	Computer-Aided Navigation Technique	Apex Location Rate	Computer-Aided Static Navigation Technique Results
Ackerman et al. 2019 [14]	In vitro	48	Clinical and radiographic	Control	11/24	Accuracy of endodontic surgery: 2.63 ± 1.38 mm
				SN	24/24	Accuracy of endodontic surgery: 1.47 ± 0.75 mm
Antal et al. 2018 [20]	CT	14	Clinical and radiographic	SN	14/14	Median angular deviation: 3.95° Median apex removal error: 0.19 mm Median osteotomy depth error: 0.37 mm
Fan et al. 2019 [15]	In vitro	42	Clinical and radiographic	Control	13/21	Accuracy of endodontic surgery: 1.92 ± 1.05 mm
				SN	20/21	Accuracy of endodontic surgery: 0.66 ± 0.54 mm
Giacomino et al. 2018 [17]	CS	3	Clinical and radiographic	SN	3/3	Apex location success rate: 100%
Pinsky et al. 2007 [16]	In vitro	110	Clinical and radiographic	Control	10/50	Accuracy of endodontic surgery in premolars: 2.47 ± 1.66 mm Accuracy of endodontic surgery in molars: 2.15 ± 1.36 mm
				SN	53/60	Accuracy of endodontic surgery in premolars: 0.63 ± 0.25 mm Accuracy of endodontic surgery in molars: 0.88 ± 0.35 mm
Popowicz et al. 2019 [18]	CS	2	Clinical and radiographic	SN	2/2	Apex location success rate: 100%
Ye et al. 2018 [19]	CS	2	Clinical and radiographic	SN	2/2	Apex location success rate: 100%

CT: clinical trial; CS: case series; SN: static navigation.

**Table 2:** Assessment of methodological quality according to the Current Research Information System (CRIS) scale.

Author/Year	Sample Preparation and Handling	Allocation Sequence and Randomization Process	Whether the Evaluators Were Blinded	Statistical Analysis	Score
Ackerman et al. 2019 [14]	Yes	Yes	Yes	Yes	4
Fan et al. 2019 [15]	Yes	Yes	Yes	Yes	4
Pinsky et al. 2007 [16]	Yes	Yes	No	Yes	3

**Table 3:** Assessment of methodological quality according to the Jadad scale.

Jadad Criteria						
Author/Year	Is the Study Described as Randomized?	Is the Study Described as Double-Blinded?	Was There a Description of Withdrawals and Dropouts?	Was the Method of Randomization Adequate?	Was the Method of Blinding Appropriate?	Score
Antal et al. 2018 [20]	NA	0	0	NA	0	0
Giacomino et al. 2018 [17]	NA	NA	NA	NA	NA	NA
Popowicz et al. 2019 [18]	NA	NA	NA	NA	NA	NA
Ye et al. 2018 [19]	NA	NA	NA	NA	NA	NA

NA: not applicable.

## Data Availability

Data sharing not applicable: No new data were created or analyzed in this study. Data sharing is not applicable to this article.
